# Supercritical Carbon Dioxide Decellularized Bone Matrix Seeded with Adipose-Derived Mesenchymal Stem Cells Accelerated Bone Regeneration

**DOI:** 10.3390/biomedicines9121825

**Published:** 2021-12-03

**Authors:** Keng-Fan Liu, Rong-Fu Chen, Yun-Ting Li, Yun-Nan Lin, Dar-Jen Hsieh, Srinivasan Periasamy, Sin-Daw Lin, Yur-Ren Kuo

**Affiliations:** 1 Division of Plastic Surgery, Department of Surgery, Kaohsiung Medical University Hospital, Kaohsiung 80756, Taiwan; cell77821@gmail.com (K.-F.L.); dr.chenrf@gmail.com (R.-F.C.); a730316@gmail.com (Y.-T.L.); yunnan1123@gmail.com (Y.-N.L.); sindawlin@gmail.com (S.-D.L.); 2 R&D Center, ACRO Biomedical Co., Ltd., Kaohsiung 82151, Taiwan; dj@acrobiomedical.com (D.-J.H.); srini@acrobiomedical.com (S.P.); 3 Department of Surgery, Kaohsiung Municipal Hsiaokang Hospital, Kaohsiung 80756, Taiwan; 4 Regenerative Medicine and Cell Therapy Research Center, Faculty of Medicine, College of Medicine, Kaohsiung Medical University, Kaohsiung 80756, Taiwan; 5 Department of Biological Sciences, National Sun Yat-sen University, Kaohsiung 80424, Taiwan; 6 Academic Clinical Programme for Musculoskeletal Sciences, Duke-NUS Graduate Medical School, Singapore 169857, Singapore

**Keywords:** supercritical carbon dioxide (scCO_2_), decellularized bone matrix (scDBM) scDBM, adipose-derived mesenchymal stem cells (ADSC), bone regeneration

## Abstract

Large bone fractures with segmental defects are a vital phase to accelerate bone integration. The present study examined the role of supercritical carbon dioxide (scCO_2_) decellularized bone matrix (scDBM) seeded with allogeneic adipose-derived mesenchymal stem cells (ADSC) as bio-scaffold for bone regeneration. Bio-scaffold produced by seeding ADSC to scDBM was evaluated by scanning electron microscopy (SEM). Rat segmental femoral defect model was used as a non-union model to investigate the callus formation in vivo. Histological analysis and osteotomy gap closure in the defect area were analyzed at 12 and 24 weeks post-surgery. Immunohistochemical expression of Ki-67, BMP-2 and osteocalcin was evaluated to assess the ability of new bone formation scDBM. ADSC was found to attach firmly to scDBM bioscaffold as evidenced from SEM images in a dose-dependent manner. Callus formation was observed using X-ray bone imaging in the group with scDBM seeded with 2 × 10^6^ and 5 × 10^6^ ASCs group at the same time-periods. H&E staining revealed ASCs accelerated bone formation. IHC staining depicted the expression of Ki-67, BMP-2, and osteocalcin was elevated in scDBM seeded with 5 × 10^6^ ASCs group at 12 weeks after surgery, relative to other experimental groups. To conclude, scDBM is an excellent scaffold that enhanced the attachment and recruitment of mesenchymal stem cells. scDBM seeded with ASCs accelerated new bone formation.

## 1. Introduction

Restoration of critical bone defects requires a sequence of processes, thus mainly depending on the integrity and biomechanical features of bone [1,2]. The most commonly used surgical procedure is bone grafting, to augment bone regeneration in orthopaedic surgery [3,4]. Annually worldwide exceeding two million bone grafting surgery were done [5]. However, non-union of bone is a common clinical issue that leads to rehospitalization and re-surgery. Currently, autografts and autologous bone is still considered as the gold standard to repair large bone defects, because they possess all the essential properties such as osteoconduction, osteoinduction and osteogenesis vital for bone regeneration [4,6,7,8]

However, the issue of limited source and donor site complications is a constrain. Bone allografts dominate the top choice for orthopaedic surgeons since they are accessible in various forms and huge amounts [9,10]. However, poor healing was detected relative to the use of autologous grafts and the risk of potential transmission of disease and other infective agents, leading to the morbidity of patients [9,10]. Therefore, there is a need for an alternative bone scaffold to facilitate bone healing and regeneration is needed for the restoration of critical bone defects.

To overcome these limitations, tremendous alternatives and possibilities have been brought by the emergence of synthetic bone substitutes during the past decades, which made bone grafts are the most promising in the orthopaedic industry. Recently, the focus has been shifted on xenogenic extracellular matrix (ECM) scaffolds, made from the decellularization of tissue and organ [11]. Xenogenic bone grafts from bovine, ovine, equine, or porcine sources are available commercially. At present, the standard protocol in the manufacture of xenogenic bone grafts is by using high-temperature sintering at 300–1300 °C, which eliminates zoonotic infectious agents, as well as those immunogenic components, in the animal bone tissues [12]. However, the high temperature destroys the inherent collagen and alters the native porous structures of the animal bones [13].

Supercritical carbon dioxide (SCCO_2_) extraction technology is a green technology with numerous advantages over the traditional bone decellularization technique, which includes natural, safe, non-toxic, non-corrosive, non-flammable, easily accessible, and cost-effective. The SCCO_2_ technology exploits supercritical fluid carbon dioxide as the extracting solvent to eradicate the fat, cells, and non-collagenous proteins from the bone. SCCO_2_ technique was employed for bone delipidation due to its excellent solvent capacity for lipids [13]. SCCO_2_ technique significantly diminishes the antigenicity in the bone decellularization process while maintaining the strength of the bone to that of native bone [13]. SCCO_2_ uses mild critical coordinates, with pressure at 7.38 MPa and temperature at 31 °C, which can easily and efficiently be accomplished and are well suited for bone graft materials (scDBM) [13].

ACRO Biomedical Co., Ltd. (Kaohsiung, Taiwan) decellularized porcine bone using SCCO_2_ technology to produce bone matrix branded as ABCcolla^®^ Collagen Bone Graft [13]. ABCcolla^®^ Collagen Bone Graft is certified by U.S. Food and Drug Administration (FDA) and Taiwan Food and Drug Administration (TFDA), the ability of the scDBM to bone healing in defect model has to be examined. In the current study, the structure of the scDBM was evaluated for the biocompatibility of adipose-derived stem cells (ASC) using a scanning electron microscope (SEM). Subsequently, we investigated the role of scDBM seeded with ASCs could promote and accelerate bone healing and regeneration by evaluating the expression of Ki-67, bone morphogenetic protein 2 (BMP-2) and osteocalcin in a rat segmental defect model.

## 2. Materials and Methods

### 2.1. Decellularizion of Porcine Bone Matrix Using SCCO_2_

Porcine bones were purchased from Tissue Source, LLC (Lafayette, IN, USA). In brief, the native bone was placed on a tissue holder, which was then inserted into a SCCO_2_ vessel system (Helix SFE Version R3U, Applied Separations Inc., Allentown, PA, USA) containing 30 mL 95% ethanol. The SCCO_2_ system was then operated at 350 bar and 45 °C for 80 min to produce scDBM [13].

### 2.2. Isolation and Culture of Adipose-Derived Stromal Cells

ASCs isolation was performed as previously described [14,15]. Briefly, groin adipose tissue from Lewis rats was collected, minced, washed, and digested with 0.4% collagenase type I for 30 min at 37 °C. The cell suspension was then centrifuged at 1500 rpm for 5 min at room temperature and the supernatant was discarded. Red blood cell was lysed with RBC lysis buffer, washed with phosphate-buffered saline, and then resuspended in a maintenance medium. Cells were then seeded in culture dishes for 24 h. Supernatant and non-adherent cells were discarded, and the attached cells were considered to be passage 0 of ASCs. Adipose-derived stromal cells were used for experiments between passages 3 and 5.

ASCs were cultured in a maintenance medium consisting of Dulbecco’s Modified Eagle Medium (Gibco) supplemented with 10% fetal bovine serum (HyClone) and 1x anti- (Gibco) at 37 °C in a humidified atmosphere with 5% carbon dioxide. ASCs were characterized by flow cytometry following positive surface staining for CD29, CD44, CD73, CD90, MHC-I, and CD106, but negative for CD31, CD34, CD45, and MHC-II. The differentiation ability of ASCs into adipocytes, osteoblasts, and chondrocytes was tested according to our previously described procedures [14,15].

### 2.3. Cell Seeding and Attachment Technique

ASCs were detached from the culture flask by 0.25% trypsin, centrifuged, and resuspended. After sterilization, scDBM are placed in a cryovial and added with 150 μL medium containing 2 × 10^6^ or 5 × 10^6^ cells and incubated at 37 °C for 2 h for cell attachment. Subsequently, cell-attached scDBM are immersed in a 1 mL complete medium. Unseeded scaffolds are used as controls, and all samples are subjected to scanning electron microscopy at day 2 after cell seeding.

### 2.4. Scanning Electron Microscopy

scDBM are fixed with 2.5% glutaraldehyde for 1 h, followed by several washing steps with distilled water. Then, samples are dehydrated through a graded series of ethanol. Finally, scDBM is subject to vacuum dehydration, fixed on metal stubs, coated with gold-palladium by sputtering (Sputter coater, SC7620, East Sussex, UK), and examined under scanning electron microscopy (SEM).

### 2.5. Animals

Male Lewis (LEW) rats weighing 400 to 450 g were used. All animals were purchased from the National Laboratory Animal Breeding and Research Center, Taipei, Taiwan. Experiments conducted were approved by the Institutional Animal Care and Use Committee at Kaohsiung Medical University (KMU IACUC-105086; 30 September 2019), Taiwan.

### 2.6. Segmental Defect Model

Rat segmental defect model was performed according to our previous studies [16,17,18]. Briefly, rats were anaesthetized with isoflurane. After opening the periosteum, a four-hole straight plate was positioned on the anterolateral femoral shaft. Proximal and distal holes were drilled and tapped with a 7-mm long bone screw. A 5-mm long, full-thickness disk of cortical and cancellous bone was resected from the middle of diaphysis with wire. A wire was passed through the hole on the fixation plate and engaged with the trabecular bone in the proximal femoral diaphysis to stabilize the fixation plate. The wound was then closed in layers.

### 2.7. Animal Experimental Design

A segmental defect model was performed on day 0. In group 1, the defect was filled with nothing as the control group. Group 2 was applied with scDBM to fill the defect. In group 3, scDBM applied to the defect was seeded with 2 × 10^6^ ASCs. In group 4, the defect is filled with scDBM seeded with 5 × 10^6^ ASCs. The animals were sacrificed and the biopsy samples were obtained at 12 weeks and 24 weeks after surgery.

### 2.8. Radiography of Osteotomy gap

Callus and osteotomy gap closure was determined by dual-energy X-ray absorptiometer in a high-resolution mode (45KVp, 4.5 s; Hologic QDR-4500A; Hologic, Waltham, MA, USA). The region of interest in the distraction zone was a 6 × 6-mm^2^ area in the center of the distracted mandible. A hemimandible specimen without distraction was used as the internal control. Bone mineral density in the mandibular tissue was expressed as g/cm^2^ (Lai et al., 2010) [16].

### 2.9. Histologic Evaluation

Biopsy specimens of bone defects were obtained at pre-determined time points (12 weeks and 24 weeks post-operation). Tissues were harvested, fixed in 10% formalin, sectioned, and stained with hematoxylin and eosin.

#### 2.9.1. Immunohistochemistry Staining

Femur sections were subject to IHC staining for Ki-67, BMP-2 and osteocalcin to detect the ability of bone formation. UltraVision Quanto Detection System (Thermo Scientific, Fremont, CA, USA) was used according to the manufacturer’s instructions. Briefly, tissue sections were incubated with UltraVision Hydrogen Peroxide Block for 10 min to stop endogenous peroxidase activity, and then UltraVision Protein Block to block nonspecific protein. The monoclonal antibodies used for immunohistochemistry were anti-Ki-67 (1:100), anti-BMP-2 (1:100) and anti-osteocalcin (1:100). Antibodies were added and incubated at 4 °C overnight. Subsequently, sections were incubated with Primary Antibody Amplifier Quanto for 10 min and then HRP Polymer Quanto for 10 min. Visualization of the specific binding was developed using DAB/Chromogen. Sections were then mounted, cleaned, cover-slipped and quantified, analyzed using a QuPath (https://qupath.github.io, accessed on 22 March 2020) the color and background estimates were applied for each IHC analysis for stain separation within QuPath using color deconvolution. By selecting a representative area containing an area of background along hematoxylin and DAB staining.

#### 2.9.2. Statistical Analysis

Data were expressed as means ± standard error. Significant differences between experimental groups were analyzed by using the t-test. A value of *p* < 0.05 was considered statistically significant.

## 3. Results

### 3.1. Surface Characteristics and Cell Adhesion of scDBM

**Figure 1:**
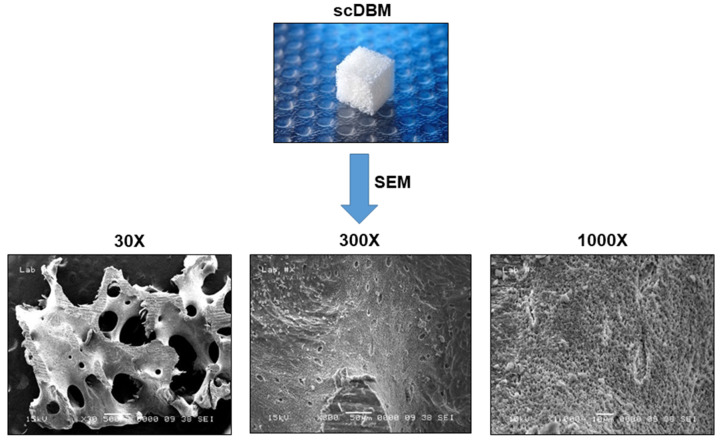
Scanning electron microscope (SEM) images of the scDBM. The highly porous and cancellous structures of the scaffold are shown in the SEM images at different magnification (30×, 300× and 1000×).

**Figure 2:**
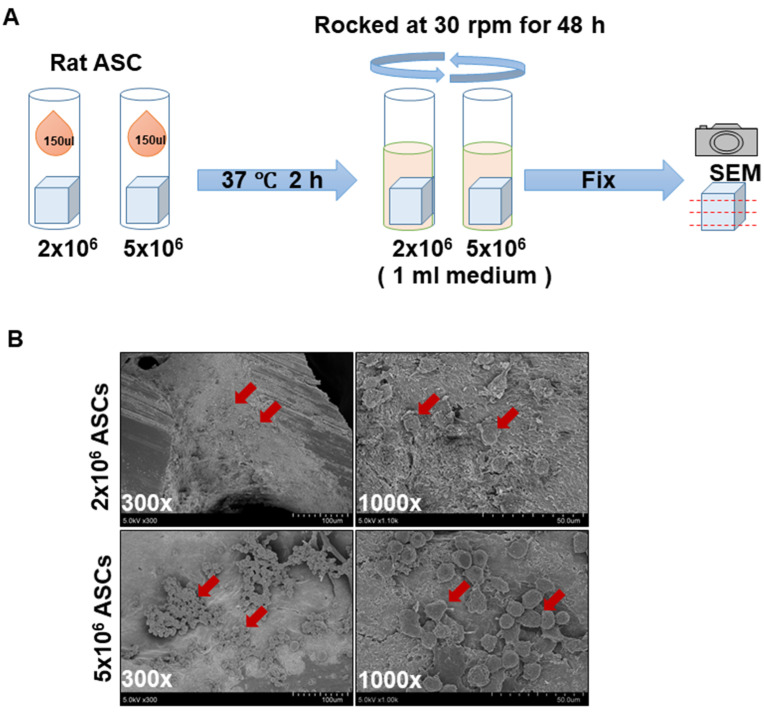
Flowchart for ASCs treatment on the scDBM. (**A**) ASCs was seeded onto the scDBM and incubated for 2 h. The scDBM was then incubated with medium for 48 h and was examined by SEM. (**B**) Cell attachment was shown (red arrows) in lower (300×) and higher magnification (1000×) SEM images (*n* = 3).

To evaluate the surface nature and cell adhesion SEM was used to examine the architectural changes of scDBM. SEM images revealed high porosity of the cancellous structure of scDBM at 30×, 300× and 1000× magnification (Figure 1). The cell adhesion ability of the scDBM after 48 h of incubation of ASC was examined using SEM (Figure 2B). SEM images displayed ASC attached to the surface of scDBM in a dose-dependent manner; while in the 2 × 10^6^ ASCs group, cells attached to the scDBM are visible, a significantly large number of ASC on the scDBM in the 5 × 10^6^ ASCs group was noticed. This result revealed high porosity and a high extent of cell attachment of the scDBM.

### 3.2. Role of scDBM and ASC on Callus Formation and Osteotomy Gap

**Figure 3:**
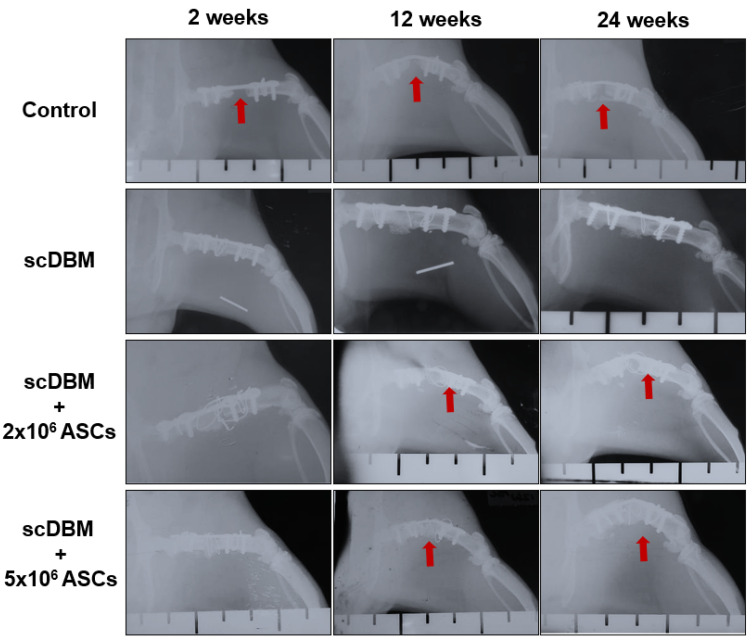
Representative radiographs of bone defect X-rays of defect at 2, 12 and 24 weeks in control, scDBM and scDBM seeded with 2 × 10^6^ and 5 × 10^6^ ASCs group. Red arrow in control group indicates defect. Red arrow in scDBM+2 × 10^6^ and 5 × 10^6^ ASCs groups indicate bone regeneration (*n* = 6).

To investigate the role of scDBM and ASC on accelerated bone regeneration and new bone formation radiographically, the scDBM and ASC is applied in a rat segmental femoral defect model. The radiograph depicts obvious segmental gaps after 2 weeks after surgery in all experimental groups (Figure 3). In the control group animals, the segmental gaps remained after 12 weeks. However, noticeable callus formation was observed in scDBM and scDBM combined with the 2 × 10^6^ and 5 × 10^6^ ASCs group after 12 weeks. After 24 weeks, although little callus formation is present at the end of the bone and the bone defect was obvious in control group animals, In contrast, excellent dense callus bridging was observed in bone defects of scDBM and scDBM combined with 2 × 10^6^ and 5 × 10^6^ ASCs group. These results strongly indicate that the scDBM promotes the formation of callus in the bone defect.

### 3.3. Role of scDBM and ASC on New Bone Formation in Bone Defects by Histology

**Figure 4:**
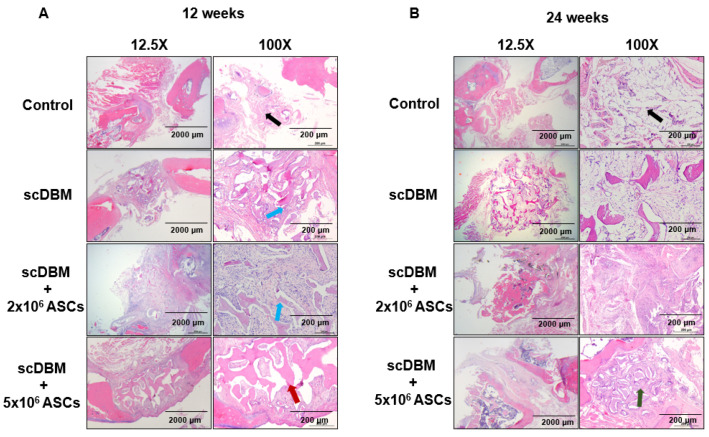
H&E staining of bone defect (**A**) H&E images showed no bone formation in the control group (@) at 12 weeks. On the other hand, the non-significant bone formation could be observed in scDBM seeded with the 2 × 10^6^ ASCs group (#), and ASCs appeared to accelerate the scDBM-promoted bone formation in the 5 × 10^6^ ASCs group (&). (**B**) At 24 weeks, bone started to bridge the gap in scDBM seeded with the 5 × 10^6^ ASCs group. Black arrow indicates fibrous tissue. Blue arrow indicates non-significant bone formation. Red arrow indicates moderate bone regeneration. Green arrow indicates new bone formation (*n* = 6). Scale bars = 200, 2000 μm.

The H&E staining revealed the bone defects are filled with fibrous tissue and muscle in the control group and no bone formation (black arrow) at 12 weeks after surgery (Figure 4A,B). In scDBM (blue arrow) and scDBM combined with 2 × 10^6^ group (blue arrow) demonstrated non-significant bone formation between segmental defects. In contrast, the scDBM combined with the 5 × 10^6^ ASCs group, the new bone formation was observed in defects at 24 weeks after surgery, and the defects are bridged by new bone formation (green arrow) (Figure 4B). These results indicate that the scDBM with ASCs promotes the callus and new bone formation in the segmental defect model.

### 3.4. Role of scDBM and ASC on the Expression of Ki-67, BMP-2 and Osteocalcin in Bone Defect

**Figure 5:**
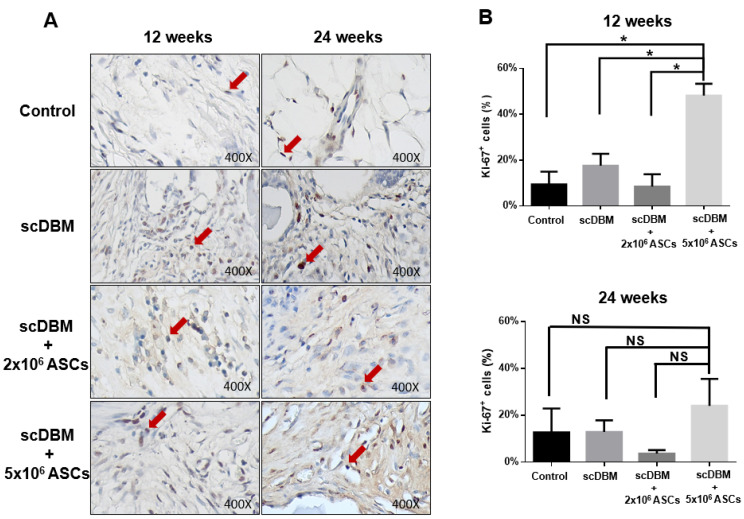
IHC staining of Ki-67 on segmental bone defects. (**A**) IHC staining of Ki-67 on bone defects at 12 and 24 weeks after the operation (400×). (**B**) Quantification scoring of Ki-67 expression. Results were expressed as mean ± SD, * *p* < 0.05 were considered statistically significant for different tests. Red arrows indicate Ki-67 expression. NS-Non-Significant (*n* = 6).

The expression of Ki-67, BMP-2 and osteocalcin in bone defect was evaluated to study the role of scDBM and ASC in cell proliferation, regeneration and new bone formation. Immunohistochemical observation showed that the expression of Ki-67 is significantly (*p* < 0.05) increased in scDBM combined with 5 × 10^6^ ASCs group at 12 weeks after surgery, compared with other groups (Figure 5A,B). After 24 weeks, no significant alterations in the Ki-67 expression were observed in all experimental groups. The inference indicates that scDBM combined with 5 × 10^6^ ASCs promotes cell proliferation of bone defects in the early stage of bone regeneration and new bone formation.

**Figure 6:**
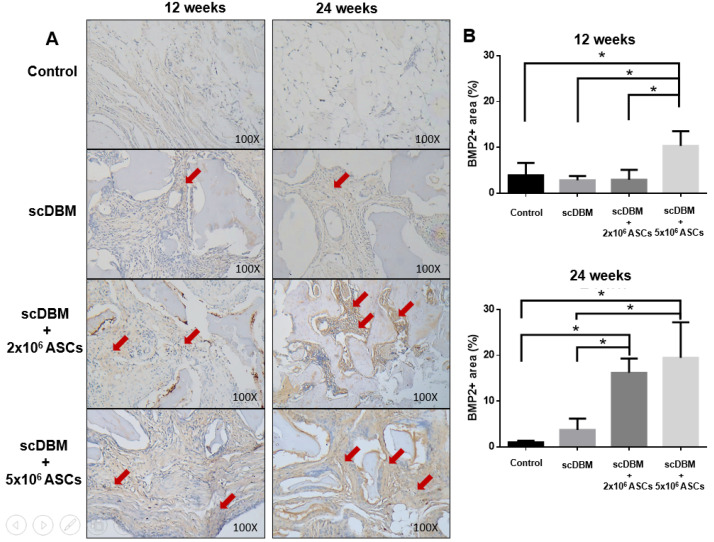
IHC staining of BMP-2 on segmental bone defects. (**A**) IHC staining of BMP-2 on bone defects at 12 and 24 weeks after the operation (100×). (**B**) Quantification scoring of BMP-2 expression. Results were expressed as mean ± SD, * *p* < 0.05 were considered statistically significant for different tests. Red arrows indicate BMP-2 expression. (*n* = 6).

The expression of BMP-2 depicted scDBM seeded with 5 × 10^6^ ASCs significantly (*p* < 0.05) increased the expression of BMP-2 at 12 weeks (Figure 6A,B). In addition, post 24 weeks, both scDBM seeded with 2 × 10^6^ ASCs and 5 × 10^6^ ASCs significantly (*p* < 0.05) increased BMP-2 expression relative to that of control and scDBM alone treated groups, indicating that seeding of ASCs to scDBM promoted the expression of BMP-2 in bone regeneration and new bone formation.

**Figure 7:**
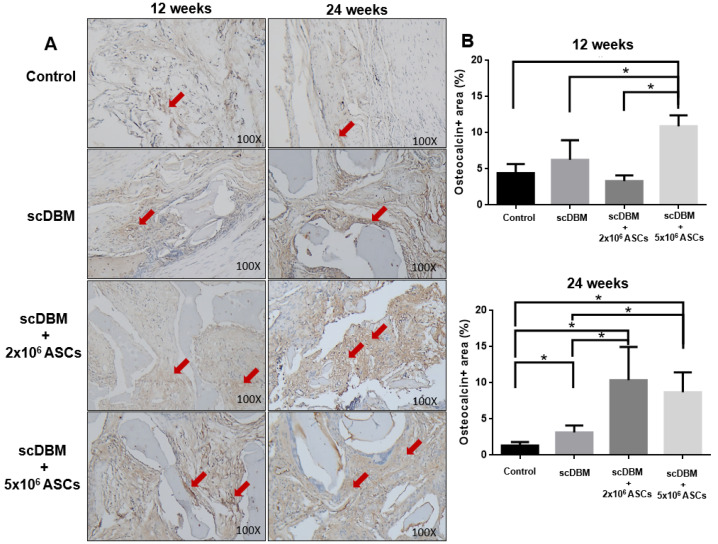
IHC staining of osteocalcin on segmental bone defects. (**A**) IHC staining of osteocalcin on bone defects at 12 and 24 weeks after the operation (100×). (**B**) Quantification scoring of osteocalcin expression. Results were expressed as mean ± SD, * *p* < 0.05 were considered statistically significant for different tests. Red arrows indicate osteocalcin expression (*n* = 6).

The expression of scDBM combined with 5 × 10^6^ ASCs significantly (*p* < 0.05) increased osteocalcin expression relative to all other experimental groups at 12 weeks (Figure 7A,B). However, after 24 weeks, scDBM alone significantly (*p* < 0.05) increased osteocalcin expression compared to that of control groups. In both scDBM seeded with 2 × 10^6^ ASCs and 5 × 10^6^ ASCs significantly (*p* < 0.05) increased osteocalcin expression relative to that of control and scDBM alone treated groups, indicating that seeding of ASCs to scDBM promoted the expression of osteocalcin in bone regeneration and new bone formation.

## 4. Discussion

Rat segmental defect model and orthopaedic injuries including complete bone fractures allow research that discovers the mechanisms of osteogenesis and evaluates the therapeutic potential of biomaterials [17]. Bone substitutes including porcine bone xenografts proved to be an outstanding grafting material for bone augmentation surgeries. Clinical usage of porcine cortical bone depicted outstanding results and hopeful future clinical uses [18]. In the present study, we used scDBM seeded with ASCs to evaluate the regenerative potential and osteoinductive nature of ASC with the presence of scDBM. We found excellent bone regeneration and new bone formation in the segmental defect model, indicating scDBM a demonstrated regenerative capability of porcine grafting material can be improved by SCCO_2_ extraction technology [19].

In the present study, scDBM seeded with ASC depicted good cell adhesion and proliferation, in which the porous nature of the scDBM was known to play a vital role in cell adhesion and proliferation. The SEM structural studies of scDBM revealed the native pore structure of porcine bone with high porosity. The porosity and pore size plays a vital role in the efficiency in cell seeding, diffusion, and mechanical strength of bone scaffold [20]. Bone remodelling was achieved by an extremely porous bone scaffold that helps vascularization, osseointegration, osteoblast and osteoclast infiltration. Reduced bone porosity restricts cell growth and infiltration for bone regeneration [21]. In the present study, the microporosity of the porcine scDBM is well conserved after the SCCO_2_. In addition, the SCCO_2_ process preserved the native structure of scDBM; it also preserved the different range and network of pores from micro- to nanosize, which are indispensable in angiogenesis and induce both bone growth and reorganization [22,23]. However, most of the bone grafts in the market are manufactured by high-temperature sintering, which alters the native pores in the bone [23], hindering the bone regeneration process.

In the current investigation, scDBM seeded with ASC depicted accelerated bone regeneration and new bone formation radiographically and histologically, the scDBM and ASC is applied in a rat segmental femoral defect model. The scDBM x-ray diffraction analysis showed a high Ca/P ratio of 1.75, indicating that the scDBM produced by the SCCO2 did not alter the chemical composition of scDBM. In addition, a high level of Ca and P stimulate osteogenesis [23,24]. The scDBMs chemical contents and structure were found to be comparable to that of human grafts. Furthermore, in vitro and in vivo biocompatibility of scDBM was found to be excellent and promising new bone formation in rabbits at 4, 12 and 24 weeks [23]. Osteochondral defects treated with scDBM in rabbits depicted good biocompatibility, healing, and bone regeneration and is effective in the regeneration of a critical defect and encouraging the new bone formation and osteoconduction [23].

ASCs are attractive for their tissue-engineering strategies in bone regeneration. In addition, ASC not only establishes outstanding cell expansion properties and osteogenic regeneration potential but also exhibits immunomodulatory properties. In addition, scaffolds are required for tissue-engineering applications to deliver cells and act as templates for tissue regeneration [25]. ASC-derived treatments have potential usage for cell-augmented bone repair approaches. However, unpredictable repair results in bone tissue engineering linked to cell population heterogeneity, variability in cell preparation, or expression of osteogenic differentiation inhibitors [26]. In the present investigation, scDBM seeded with ASC depicted accelerated bone regeneration and new bone formation in the rat segmental femoral defect model.

In the current investigation, scDBM seeded with ASC depicted increased expression of Ki 67 thus leading to accelerated bone regeneration and new bone formation in a rat segmental femoral defect model. Ki67 are frequently used to study cellular proliferation during bone regeneration. The cell proliferation marker Ki 67 is expressed by proliferating bone cells in the late G1, S, and G2/M phases of the cell cycle. The nuclear localization of Ki 67 and its specific association with the cell cycle shows its significance in the regulation of cell division during bone regeneration [27].

In the present study, scDBM seeded with ASC depicted increased expression of BMP 2 and osteocalcin, thus leading to accelerated bone regeneration and new bone formation in a rat segmental femoral defect model. BMP2 is up-regulated in all the osteogenic processes. In bone healing, BMP2 is expressed in pre-hypertrophic chondrocytes, osteoblasts, osteocytes, and vascular cells [28]. BMP2 is the crucial component of the BMP signalling cascade in human fracture callus. A noteworthy decrease in BMP2 expression in the cartilaginous areas of human nonunion fractures relative to normal healing fractures [29]. BMP2 is likely to play a vital role in fracture repair, specifically at the early stage of fracture healing [30]. The role of BMP2 in bone formation and fracture repair has been broadly studied by in vitro, ex vivo and in vivo studies [31]. Osteocalcin is involved in the recruitment, differentiation and maturation of osteoclasts [32]. Scaffolds prepared from the decellularized extracellular matrix of porcine cancellous bone increased the expression of osteocalcin and supported MSC differentiation [33]. BMP2 is an effective osteoinductive cytokine that plays a vital role during bone regeneration and repair. BMP2 acts in the extracellular environment, sulfated polysaccharides attached covalently to syndecan and also non-covalently to fibronectin fibers that bind BMP2 through a heparin-binding domain and regulate its bioactivity [34].

Phosphorene are two-dimensional materials; phosphorus derivative graphene oxide nanomaterials demonstrate optical and mechanical properties, electrical conductivity, excellent biocompatibility, and good biodegradation. Therefore, they used to promote bone regeneration [35]. However, ABCcolla^®^ Collagen Bone Graft is SCCO2 decellularized porcine bone with 3D natural bone pores for the stem cells to reside and proliferate, which helps in enhanced neovascularization, osteointegration, excellent biocompatibility, bioresorbable and osteoconductive [13,23]. The comparative analysis of other bone substitutes used in bone repair and regeneration and their functions are shown in Table 1. The scDBM was found to involve in bone regeneration with mechanical stiffness. The efficacy of scDBM on bone regeneration was assessed in dog mandibular extraction socket and revealed significantly greater stiffness treated sites in the biomechanical analysis [13,23]. The scDBM was found to be a strong implant covering over the orbital defect with a low complication rate, low infection rate, low cost, high biocompatibility and high osteoconductive properties [36]. BMP2 induces osteoblast differentiation, and the osteoblast protein osteocalcin production in osteoblastogenesis. Bone calcification is mediated by the expression of BMP2 and the BMP2 receptor–Smad signaling pathway. BMPs are a superfamily of transforming growth factor-beta and secretory growth factor, possess osteogenic actions and play important roles in bone formation. BMP2, GEP, and Jun B also constitute a regulatory loop during osteoblast differentiation extending the interplay networks among these molecules. BMP2 contribute to a regulatory feedback loop in which the transactivation of IRE1a expression by Jun B leads to a BMP2-mediated suppression of osteoblastogenesis. Proinflammatory cytokines, including IL-1α, TNF-α and IL-17, exhibit osteoclastogenic properties, whereas others, including IL-6, may produce stimulatory and suppressive actions on osteoclasts [37,38]. Bone healing strongly differs between species, within breeds of the same species, and also at different locations within the same animal. Our evaluation was limited to radiographic and histology, the assessment of biomechanical features of the healing process might be crucial data, which we planned for our future detailed study.

## 5. Conclusions

The scDBM combined with ASCs enhanced callus formation in a segmental femoral defect. The possible mechanism of scDBM might be modulation in the expression of BMP 2 and osteocalcin, thus leading to accelerated bone regeneration and new bone formation in a rat segmental femoral defect model. The research results indicate that the scDBM scaffold is an excellent biomaterial for bone tissue repair. We describe a biomimetic bone tissue engineering approach that summarizes certain features of the natural endochondral cascade in bone defect regeneration. Implantation of scDBM seeded ASCs stimulated endochondral ossification for significant bone regeneration. The scDBM seeded ASCs system is of clinical relevance for segmental defect bone regeneration, and our findings advance the present thoughts in the field of developmental bone engineering.

## Figures and Tables

**Table 1:** Bone substitutes for bone repair and regeneration and their functions.

Product Name	Material Nature	Function	Uses
ABCcolla^®^ Collagen Bone Graft	SCCO_2_ decellularized porcine bone	Enhanced neovascularization, Osteointergration, Excellent biocompatibility, Bioresorbable and Osteoconductive	Void filling, Guided bone regeneration, Maxillofacial surgery, Orbital for reconstruction
Collagraft	A mixture of tricalcium phosphate, bovine collagen, and hydroxyapatite	Bioresorbable and osteoconductive	Use for the treatment of long bone fracture and void filling
DynaGraft	Demineralized bone matrix	Heat sensitive copolymer, limited osteoinduction	Dental bone graft substitute
CopiOs (Zimmer Biomet) Bone Void Filler	Calcium phosphate, dibasic (DICAL), and highly purified Type I bovine collagen	DICAL provides significantly more calcium and phosphate ions at equilibrium than either β-TCP or HA	Scaffold for the growth of new bone
Osteograf	Ceramic	Osteoconductive, limited osteoinductive when mixed with bone marrow	Bone void filler
NovaBone	Bioactive glass	Osteoconductive, limited osteoinductive when mixed with bone marrow	Filling surgical or traumatic bone gaps
Hard tissue replacement (HTR)	Polymethyl methacrylate (PMMA)	Good strength, durable, and surface osteoconductive	Craniofacial reconstruction
OSIQ (Kyeron)	Fully synthetic ultrapure nano-hydroxyapatite	Biodegradable	Filling or reconstruction of small and medium bone defects
